# Gender Differences in the Epidemiology of Alcohol Use and Related Harms in the United States

**DOI:** 10.35946/arcr.v40.2.01

**Published:** 2020-10-29

**Authors:** Aaron M. White

**Affiliations:** 1 National Institute on Alcohol Abuse and Alcoholism, National Institutes of Health, Bethesda, Maryland

**Keywords:** alcohol use disorder, sex, brain, development, stress, mental health, alcohol

## Abstract

Over the past century, differences in alcohol use and related harms between males and females in the United States have diminished considerably. In general, males still consume more alcohol and experience and cause more alcohol-related injuries and deaths than females do, but the gaps are narrowing. Among adolescents and emerging adults, gaps in drinking have narrowed primarily because alcohol use among males has declined more than alcohol use among females. Among adults, alcohol use is increasing for women but not for men. Rates of alcohol-related emergency department visits, hospitalizations, and deaths all have increased among adults during the past two decades. Consistent with the changing patterns of alcohol use, increases in these outcomes have been larger for women. Recent studies also suggest that females are more susceptible than males to alcohol-induced liver inflammation, cardiovascular disease, memory blackouts, hangovers, and certain cancers. Prevention strategies that address the increases in alcohol consumption and unique health risks for women are needed.

## INTRODUCTION

Alcohol consumption has long been a male-dominated activity. Globally, men consume more alcohol and account for more alcohol-related harms to self and others than women do. In 2016, 54% of males (1.46 billion) and 32% of females (0.88 billion) age 15 and older worldwide consumed alcohol.^1^ Alcohol caused roughly 3 million deaths (5% of all deaths) that year, including 2.3 million deaths for men (8% of deaths) and 0.7 million deaths for women (3% of deaths). Although gender gaps in alcohol use seemingly are universal, the size of the gaps varies between countries and their respective cultures, from a male to female ratio for current drinking of 1:1 in New Zealand and Norway to 12.3:1 in India.^1^–^3^ Large variations between countries suggest that culturally prescribed gender roles, above and beyond physiological sex differences, are central in shaping gender-specific drinking patterns.^4^

In the United States, more males than females drink each year (68% males, 64% females). Males drinkers tend to drink more often and more heavily than females do,^5^ consuming nearly three times as much pure alcohol per year (19.0 liters for males, 6.7 liters for females).^1^,^6^ Males also are more likely to be arrested for driving under the influence of alcohol (DUI),^7^ treated in emergency departments and hospitals for alcohol-related harms,^8^–^10^ and to die from alcohol-related causes.^11^ In addition, more males (7%) than females (4%) are diagnosed with an alcohol use disorder (AUD) each year. Among those with AUD, roughly similar percentages of males (9%) and females (9%) receive treatment.^6^ Research examining harms experienced due to another person’s drinking suggests women are more likely than men to suffer consequences as a result of alcohol use by a spouse/partner/ex-partner (4.2% vs. 1.8%) or a family member (5.6% vs. 3.7%).^12^,^13^

## NARROWING GENDER GAPS

**Figure 1:**
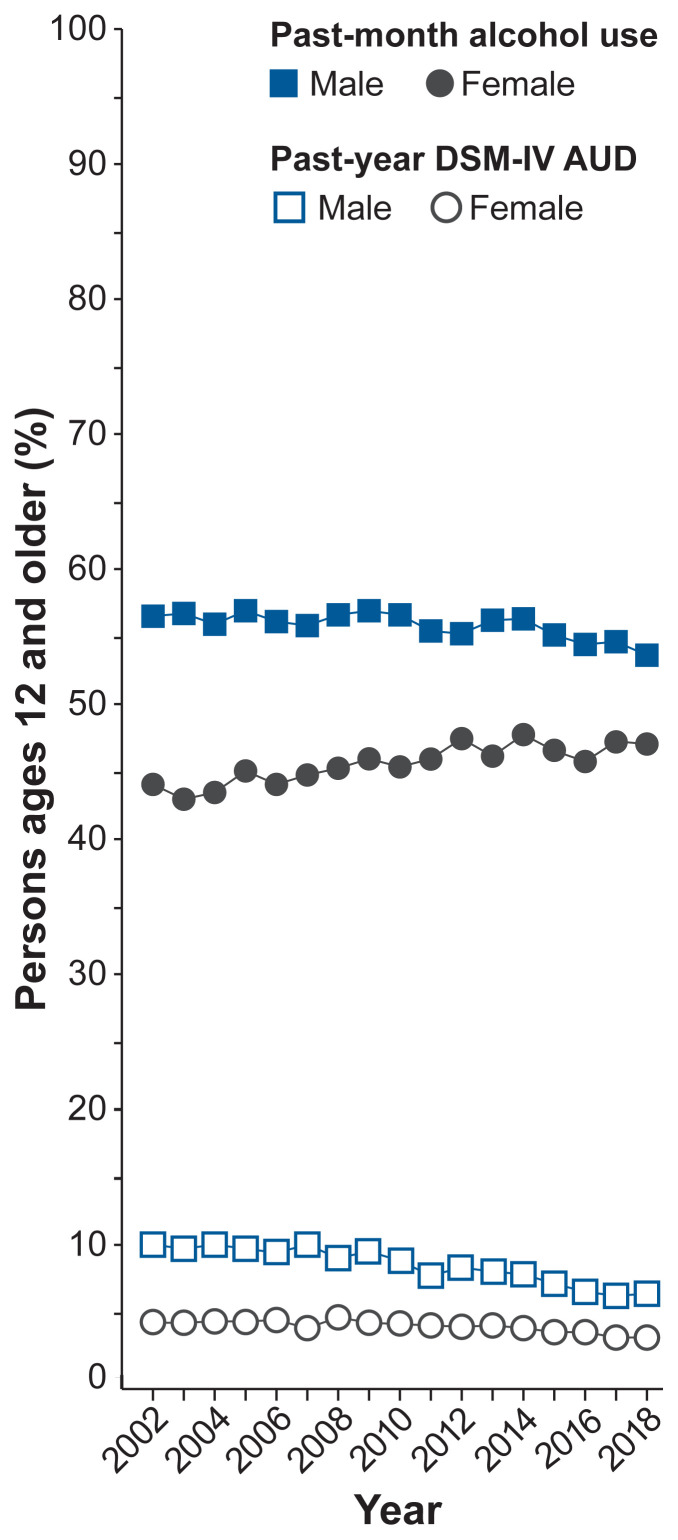
Narrowing gender gaps in the prevalence of past-month alcohol use and past-year DSM-IV AUD between females and males age 12 and older using data from NSDUH 2002–2012 Gender gaps narrowed for both measures, primarily due to increases in alcohol use among females and smaller declines in AUD among females than males. *Source:* White et al., 2015.^5^

Although males still outpace females for most alcohol-related measures, the gaps are narrowing^5^,^14^ (see Figure 1). In the 85 years since the end of Prohibition, drinking habits of males and females have converged. For cohorts born near 1900, males outnumbered females roughly 3:1 for measures of alcohol consumption (e.g., prevalence, frequency) and problematic drinking (e.g., binge drinking, early-onset drinking). Many of these ratios are closer to 1:1 today, and the differences continue to become smaller (see the box **Summary Statistics on Female and Male Alcohol Use and Outcomes in the United States** and Figure 1).^14^ An analysis of six different national surveys between 2000 and 2016 suggests that the number of women age 18 and older who drink each year increased by 6% but decreased by 0.2% for men, and the number of women who binge drink increased by 14% but by only 0.5% for men.^15^ As this article explores, gender gaps are narrowing for different reasons among adolescents and emerging adults relative to adults. Specifically, alcohol use is declining faster for adolescent and emerging adult males than for females, whereas gaps are narrowing among adults because of increases in drinking by women but not by men.^15^,^16^

**Summary Statistics on Female and Male Alcohol Use and Outcomes in the United States**

**Table:** 

Drinking patterns
Female drinkers consume about one-third as much total pure alcohol per year as male drinkers (6.7 liters for females, 19.0 liters for males).^1^ Alcohol use among people age 12 and older: *Lifetime*—82% male, 78% female; *Past year*—68% male, 62% female; *Past month*—55% male, 46% female; *Binge* (4+/5+)* *past month*—29% male, 20% female^28^
DSM-IV AUD† (alcohol abuse or dependence) age 12 and older
Past-year AUD—males, 9.2 million (7%); females, 5.3 million (4%)^28^ Percentage who needed and received treatment for DSM-IV alcohol abuse or dependence—males, 9%; females, 9%^28^
Overall deaths
In 2017, 72,558 death certificates listed alcohol as a factor (18,072 females and 54,486 males).^64^ Using death certificates and estimates, the Centers for Disease Control and Prevention calculated that 93,296 people died from alcohol-related causes each year between 2011 and 2015 (26,778 females and 66,519 males).^11^ The World Health Organization reported that excessive drinking accounted for roughly 3 million deaths (5% of all deaths) worldwide, including 2.3 million deaths for men (8% of deaths) and 0.7 million deaths for women (3% of deaths).^1^
Cirrhosis deaths
In 2017 there were 44,478 deaths due to cirrhosis and 50% (22,246) were caused by alcohol (15,470 deaths among males; 6,776 deaths among females).^10^ Overall, the rate of death from alcohol-related cirrhosis is more than twice as high for men (9.7 per 100,000) than for women (4.1 per 100,000).^10^
Driving under the influence
More men (10%) than women (5%) reported driving under the influence of alcohol (DUI) in the past year in 2017.^19^
Gender gaps are narrowing
Differences are shrinking in drinking patterns, AUD, hospitalizations, emergency department visits, DUI, liver disease, and deaths.^5^,^14^–^16^,^31^

* **Binge drinking:** Defined as four or more drinks on an occasion for females and five or more drinks on an occasion for males (4+/5+).

† **AUD:** According to criteria for alcohol abuse and alcohol dependence in the fourth edition of the *Diagnostic and Statistical Manual of Mental Disorders* (DSM-IV).

## ADOLESCENTS

Alcohol use, like other drug use, becomes more likely as young people enter and progress through adolescence, which encompasses the second decade of life or more.^17^ Data from the 2018 National Survey on Drug Use and Health (NSDUH) suggest that, by age 12, approximately 1 in 100 (1%) adolescents report consuming alcohol in the previous month.^6^ The prevalence increases to nearly 1 in 4 (23%) by age 17. Racial, ethnic, and gender differences in alcohol use also emerge during this period (see Table 1). Among students ages 12 to 17, past-month alcohol use is reported by 12% of White students, 9% of Hispanic or Latino students, 8% of American Indian or Alaska Native students, 6% of Black or African American students, 6% of Asian students, and 11% of students of two or more races.^6^ Although more boys (19%) than girls (13%) start drinking before age 14, girls who begin drinking in early adolescence have a shorter time period between first drink and first episode of binge drinking.^6^,^18^ In contrast, when drinking starts at age 15 or later, males progress more quickly to binge drinking.

Data from the 2018 NSDUH (see Table 1) suggest that 5% of adolescents (5% of females and 5% of males) ages 12 to 17 engage in binge drinking each month, defined as having four or more drinks on an occasion for females or five or more on an occasion for males.^19^ The National Institute on Alcohol Abuse and Alcoholism defines binge drinking as reaching a blood alcohol concentration (BAC) of 0.08%, the legal limit for operating a motor vehicle for adults age 21 and older, which takes about four drinks in 2 hours for women or five drinks in 2 hours for men (https://www.niaaa.nih.gov/alcohol-health/overview-alcohol-consumption/moderate-binge-drinking). It should be noted that, for most teens, drinking four or five drinks can produce a BAC well beyond 0.08%. When typical body weights of adolescents are taken into consideration, the number of drinks needed to reach a BAC of 0.08% is closer to three standard drinks within a 2-hour period for girls ages 9 to 17 and boys ages 9 to 13, four drinks for boys ages 14 to 15, and five drinks for boys ages 16 to 17.^20^ Thus, it is likely that studies that assess binge drinking among adolescents by using the criteria of four or more drinks for girls and five or more for boys, or in some cases a five-drink threshold for both males and females,^21^ underestimate the extent of potentially dangerous alcohol consumption, particularly among young females.

**Figure 2:**
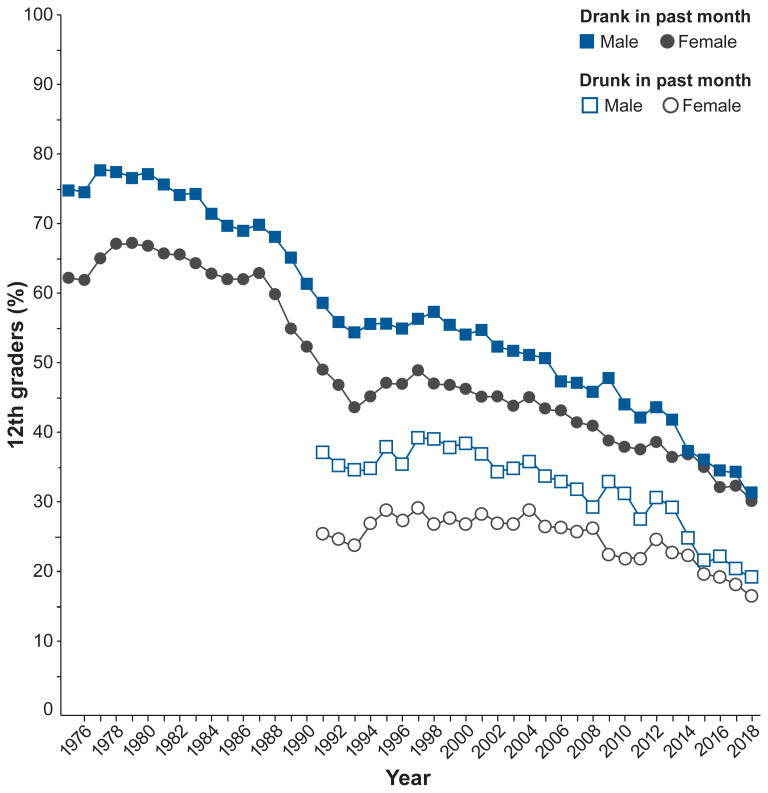
Past-month alcohol use from 1975 to 2018 and past-month drunkenness from 1991 to 2018 among 12th graders Alcohol use and drunkenness declined more for young males than for young females, leading to disappearing gender gaps in 12th grade. *Source:* Adapted from Johnston, 2019.^22^

Alcohol consumption, including binge drinking, declined significantly among adolescents since the beginning of the new millennium. Between 2002 and 2018, past-month alcohol use by adolescents ages 12 to 17 decreased from 18% to 9% and binge drinking declined from 11% to 5%.^19^ The declines in drinking were much larger for young males than for young females, leading to significant narrowing of long-established gender differences in alcohol use among adolescents. Until recently, by 10th grade, young males reported higher levels of alcohol use and binge drinking than females. By 12th grade, the differences were quite large and remained so throughout adulthood. These gender differences are disappearing and have reversed for some measures. According to data from the Monitoring the Future (MTF) study, in 1991, 46% of males and 40% of females in 10th grade reported drinking in the past month. By 2018, levels declined significantly for both and the gender gap reversed, with 22% of females reporting alcohol use in the past month compared to 17% of males.^22^ Among 12th graders, in 1991, 58% of males and 49% of females drank in the month before the survey. In 2018, past-month alcohol use was equally prevalent among males (30%) and females (30%). Gender differences in self-reported past-month drunkenness among 12th graders also narrowed considerably between 1991 (37% males, 25% females) and 2018 (19% males, 16% females), as shown in Figure 2.

Smaller declines in alcohol use and drunkenness by girls are troubling for several reasons. Evidence suggests that levels of anxiety and depression are increasing among adolescents, particularly females,^16^,^23^ and it appears that females, in general, are more likely than males to drink to cope.^24^,^25^ Drinking to cope is associated with faster progression of alcohol use and a higher incidence of alcohol-related harms.^26^ The percentage of adolescents who report drinking alone on their last drinking occasion also is increasing, and more so for girls than boys.^6^ In a longitudinal study, more episodes of drinking alone during adolescence predicted a larger number of AUD symptoms during emerging adulthood.^27^

Roughly 1 in 9 students, including 10% of females and 13% of males, drop out of school by 12th grade. Compared to teens who stay in school, those who drop out are more likely to drink and/or use other drugs. In 2014, approximately 1 in 3 (32%) students who dropped out (37% males, 26% females) reported binge drinking compared with 1 in 5 (26% males, 16% females) 12th-grade students in school.^28^ Males and females who drop out also are more likely to smoke cigarettes, use marijuana, and misuse prescription medications.^6^ Effective prevention strategies are needed to address alcohol and other drug use in this population.

## EMERGING ADULTS

**Figure 3:**
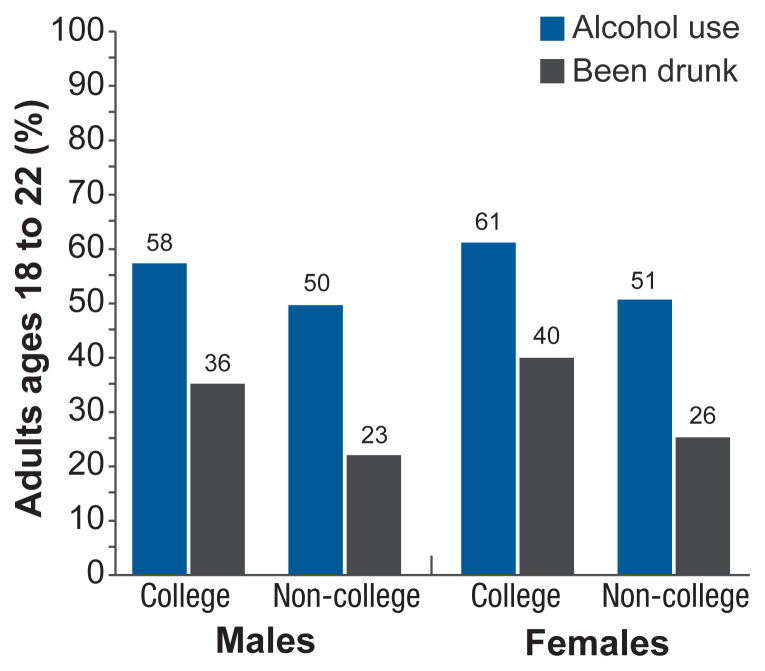
Past-month alcohol use and drunkenness among emerging adults (ages 18 to 22) based on college status Both measures are declining more for emerging adult males than for emerging adult females, leading to disappearing gender gaps. *Source:* Adapted from Schulenberg et al., 2019.^21^

Over the past few decades, alcohol use declined among emerging adults, although the declines were smaller than those seen among adolescents.^21^ Gender gaps narrowed as well. Roughly 40% of people ages 18 to 24 are enrolled in college. Historically, male college students were more likely to drink and did so more heavily than female college students, and college students drank far more than their peers not enrolled in college. Gender differences among college students have disappeared for some measures. For instance, in 1953, 80% of males and 49% of females in college reported having been drunk at some point in their lives.^29^ In 2014, 69% of both males and females in college reported having been drunk at some point in their lives.^30^ Differences in alcohol use among college students and their non-college peers are shrinking as well. According to data from the MTF study, between 1980 and 2018, the prevalence of binge drinking—in this case having five or more drinks on an occasion in the previous 2 weeks for both males and females—declined among males in college from 52% to 32% and among males not in college from 54% to 25%.^21^ The declines were smaller for females. The prevalence declined for females in college from 36% to 27% and for females not in college from 29% to 25%. For past-month alcohol use and reports of being drunk, the gender gaps reversed, with females both in and outside of college exceeding the levels among their male counterparts (see Figure 3).^22^ In 2018, 61% of females in college and 51% of females not in college reported past-month drunkenness, compared to 58% of males in college and 50% not in college. These shifts are remarkable given the long history of heavier alcohol use among young adult males than females.

## ADULTS

Despite declines in alcohol use among adolescents and emerging adults, the prevalence of alcohol use, binge drinking, and the number of drinking days in the past month increased among all females age 12 and older between 2002 and 2012.^5^ These measures did not increase among males, leading to narrowing gender gaps. Figure 1 shows narrowing gender gaps in past-month alcohol use and past-year AUD—according to criteria for alcohol abuse and alcohol dependence in the fourth edition of the *Diagnostic and Statistical Manual of Mental Disorders* (DSM-IV). An examination of alcohol measures among adults age 18 and older in six national surveys showed increases in past-year alcohol use and binge drinking among females between 2000 and 2016, with no increases for males.^15^ The prevalence of alcohol consumption and binge drinking did not increase for young adults ages 18 to 29, but increased for all adults age 30 and older, with the biggest increases occurring among people beyond age 50.

Several studies suggest that alcohol use and related harms are increasing among older people as the baby boomer cohort (now ages 55 to 75) ages. As with adults as a whole, the increases in alcohol use among older drinkers have been larger for women than for men.^14^,^31^,^32^ Between 2005 and 2014, past-month binge drinking among adults age 50 and older increased more for women (6% to 9%) than for men (20% to 22%).^31^ During that time period, the prevalence of past-year AUD also increased more for women age 50 and older (1.3% to 2.4%) than for men in that age group (5.0% to 5.1%). Similarly, data from the National Health Interview Surveys suggest that, between 1997 and 2014, the prevalence of past-month drinking among adults aged 60 and older increased more for women than for men, and the prevalence of binge drinking in this age group increased for women only.^32^ Consistent with narrowing gender gaps in alcohol use among older drinkers, between 2006 and 2014, the rates of emergency department (ED) visits related to both acute and chronic alcohol consumption increased more for women than men among those ages 55 to 64.^8^

## SEXUAL ORIENTATION

Sexual orientation influences drinking patterns and alcohol-related outcomes for males and females.^33^–^35^ In the 2018 NSDUH, past-month binge drinking (four or more drinks for females and five or more drinks for males) was reported by 26% of respondents who identified as heterosexual, 33% who identified as lesbian or gay, and 37% who identified as bisexual.^6^ Data from the National Epidemiologic Survey on Alcohol and Related Conditions III suggest that lesbians and bisexual women are twice as likely as heterosexual women to engage in binge drinking each year (lesbian 49%, bisexual 59%, heterosexual 26% )^35^ (see Table 2). Lesbians and bisexual women also are more likely than heterosexual women to consume 12 or more drinks on an occasion—three times the standard binge threshold for women—in the past year (lesbian, 8%; bisexual, 8%; heterosexual, 3%). Consuming 12 or more drinks is potentially lethal. In a study based on data from the 2000 National Alcohol Survey, lesbians were nearly 11 times more likely, and bisexual women eight times more likely, than heterosexual women to report negative social consequences from drinking.^34^,^36^ Among emerging adults ages 18 to 25, 8% of heterosexual women reached criteria for DSM-IV AUD in the previous year, compared to 15% of lesbians and 10% of bisexual women.^6^ Alcohol use does not decline as much with age among sexual minority women relative to heterosexual women.^37^ Overall, the influence of sexual orientation on alcohol use and related outcomes appears to be greater among women than among men.^38^,^39^

## PREGNANCY

In 1973, a paper by Jones and Smith detailed a syndrome involving facial dysmorphology, growth retardation, and central nervous system dysfunction in children exposed to alcohol in the womb.^40^ Since then, our understanding of the effects of alcohol on embryonic and fetal development has advanced greatly, yet alcohol use during pregnancy remains a significant public health concern. An examination of data from the Behavioral Risk Factor Surveillance Survey suggests that from 2015 to 2017, 12% of pregnant women drank alcohol and 4% engaged in binge drinking in the previous month.^41^ The average frequency of binge drinking was five times per month and the average number of drinks per binge was six.

A report using data from NSDUH suggests that past-month alcohol use did not decline between 2002 and 2017 for non-pregnant women ages 18 to 44 (from 57% to 58%) but did decline for pregnant women in this age group (from 13% to 10%).^42^ Between 2002 and 2014, past-month binge drinking—in this case, five or more drinks on an occasion—increased for non-pregnant women (24.9% to 26.6%) but declined for pregnant women (4.7% to 2.9%).^42^ Risk factors associated with alcohol use or binge drinking during pregnancy include the use of other substances, meeting DSM-IV criteria for AUD, depression, and being unmarried. An examination of NSDUH data averaged between 2001 and 2011 suggests that alcohol use during pregnancy tends to decline abruptly after the first month as women discover they are pregnant. Among pregnant women, 42% reported drinking in the first month, declining to 17% in the second month and 8% in the third month. For binge drinking, prevalence declined from 20% in the first month of pregnancy to 9% in the second month and 3% in the third month.^43^ Monthly declines were much smaller for women who met criteria for DSM-IV alcohol dependence in the previous year.

Despite declines in drinking during pregnancy, the fact that roughly 1 in 10 pregnant women still drink each month is concerning.^44^ A recent estimate suggests that the prevalence of fetal alcohol spectrum disorder (FASD) in the United States is 1% to 5%.^45^ A prospective study of roughly 31,000 women found that birth weight in newborns was reduced even when the mother’s alcohol intake was limited to an average of one drink per day (14 grams of alcohol).^46^ Drinking even 3.5 standard U.S. servings of alcohol (14 grams each) per week is associated with lower IQ scores in offspring at age 8, particularly if they have one of four genetic variants in alcohol-metabolizing genes.^47^ Alcohol exposure during the first trimester appears to be particularly detrimental, but even low to moderate levels of alcohol exposure throughout pregnancy are associated with morphological, cognitive, and motor deficits.^44^,^48^ It should be noted that recent studies raise the possibility that alcohol use by the father before conception also might influence fetal development and later alcohol use.^49^

## HEALTH EFFECTS

As patterns of alcohol use by girls and women changed over the past few decades, so did our knowledge about the potential health consequences faced by female drinkers. Research suggests that, although women tend to drink less than men, a risk-severity paradox occurs wherein women suffer greater harms than men at lower levels of alcohol exposure.^50^ For instance, men in the military drink more heavily than women in the military, yet women are at greater risk of DSM-IV alcohol dependence and lost productivity.^51^ The number of drinks needed to feel drunk is one-third lower among women (four drinks) than men (seven drinks), probably relating to lower average body weights and less total body water in women.^52^ Despite drinking less often and less heavily than males, roughly similar percentages of female and male drinkers in college report having experienced at least one alcohol-induced memory blackout in the past 2 weeks (10% females, 9% males),^53^ in the past 6 months (22% females, 17% males),^54^ and in the past year (29.2% females, 28.8% males).^55^ Females with AUD perform more poorly than males with AUD on a variety of cognitive tasks, even with fewer years of AUD.^56^ Research suggests that women have faster progression of AUD than men and are at greater risk than men for alcohol-induced hangovers, liver inflammation, cardiovascular diseases, and certain cancers.^11^,^57^–^60^ Compared with their male counterparts, women with alcoholic liver disease have a more rapid progression to fibrosis that persists after abstinence from alcohol.^61^ The Million Women Study in the United Kingdom, which included more than 28,000 women with breast cancer, suggests that every 10 grams of alcohol consumed per day (less than one standard 14-gram U.S. serving) was associated with a 12% increase in the risk of breast cancer.^62^ Because women reach higher blood alcohol levels than do men of comparable weight, their body tissues are exposed to more alcohol and acetaldehyde, a toxic metabolite of alcohol, with each drink.^63^

## MEDICAL EMERGENCIES AND DEATHS

Long-standing gender differences in alcohol-related medical emergencies and deaths are narrowing. Alcohol-related hospitalizations and ED visits increased over the past few decades, and rates increased more for women.^8^,^10^,^64^ Although men still account for the majority of these events, women are catching up. For instance, between 2006 and 2014, the number of ED visits involving alcohol increased from 2,132,645 to 3,366,477 for men (a 58% increase) and from 947,173 to 1,609,320 for women (a 70% increase).^8^

Between 1999 and 2017, nearly 1 million people died from alcohol-related injuries, overdoses, and diseases in the United States.^64^ The number of such deaths more than doubled from 35,914 per year to 72,558 per year, and the rate increased 51%, from 17 to 26 per 100,000. Males accounted for the majority (76%) of alcohol-related deaths over the years (721,587 males, 223,293 females). However, a steeper increase was observed for females (136% in numbers, 85% in age-adjusted rates) than for males (93% in numbers and 39% in rates). Over the years, rates of alcohol-related deaths were highest for males and females in the age range of 45 to 74, but the biggest increase in rates occurred among young adults ages 25 to 34 for both genders. Deaths related to injuries and overdoses increased significantly for females ages 16 to 20 but did not change for males. Although alcohol-related mortality increased each year for non-Hispanic White males and females, there were initial declines early on for several groups. By the end of the study period, deaths were increasing in all racial and ethnic groups for both males and females in nearly every age group.

## DRIVING UNDER THE INFLUENCE

Driving under the influence of alcohol (DUI) declined over the past few decades, but the rates of decline were greater for males than females.^65^ For instance, Schwartz and Davaran reported that, between 1990 and 2007, rates of arrests for DUI declined by 32% for males (from 2,019 to 1,033 per 100,000) but by only 5% for females (from 306 to 275 per 100,000).^66^ The authors suggested that the smaller decline among females might be partly related to changes in DUI enforcement practices. Schwartz observed a similar narrowing of the gender gap in DUI arrests due to steeper declines for males than females between 1982 and 2004.^67^ Reilly et al. reported that the percentage of DUI arrests involving female drivers increased in California from 11% in 1989 to 24% in 2012.^68^ Further, the percentage of female clients attending a DUI program in southern California increased from 28% in 2009 to 31% in 2014. Among male drivers who died in car crashes, the percentage of crashes in which the driver had a BAC of 0.08% or greater decreased from 25% in 2008 to 21% in 2017. In contrast, there was a small increase in the percentage of female drivers in fatal crashes with BACs greater than 0.08%, from 13% to 14%.^69^ Overall, it appears that differences in the prevalence of DUI arrests and fatalities between males and females are becoming smaller.^70^

## HARMS TO OTHERS

Alcohol consumption by an individual often leads to harms to others, also known as secondhand harms.^12^,^71^,^72^ Traffic crash injuries and fatalities are well-known secondhand harms caused by another person’s alcohol use, but there are more. A recent study by Nayak and colleagues utilized data from the 2015 National Alcohol’s Harms to Others Survey, which asked respondents about secondhand harms such as having property vandalized or damaged, being harassed or assaulted, or experiencing financial troubles.^12^ The findings suggest that roughly 1 in 5 adults in the United States experiences harm due to someone else’s alcohol use each year. This includes 21% of adult women and 23% of adult men. Women and men under age 25, those who were unmarried, and those who drank excessively, were more likely to report experiencing secondhand harms. Women more often than men reported harm related to aggression on the part of an alcohol-consuming spouse, partner, ex-partner, or family member. Men were more likely to report harm because of a stranger’s drinking. Additional research on secondhand harms from alcohol use could be helpful for elucidating gender differences in the risk for alcohol-related consequences.

## SUMMARY

For at least a century, differences in the prevalence and amount of alcohol consumption between males and females in the United States have been narrowing.^73^–^76^ As a result, so have rates of alcohol-related harms, including DUIs, ED visits, hospitalizations, and deaths. Although men still account for more total alcohol consumption and the negative outcomes that follow, the gaps are slowly disappearing. In fact, among adolescents and emerging adults, females are now more likely to report drinking and getting drunk in the past month than their male peers for the first time since researchers began measuring such behaviors.

Importantly, it is not the case that women in the U.S. are simply drinking more like men. Instead, women and men appear to be moving toward one another in terms of drinking patterns and harms. Among adolescents and emerging adults, narrowing gaps are being driven primarily by faster declines in alcohol use by males than females. Among adults, gaps are narrowing primarily because women are drinking more while men are either drinking less or maintaining their levels.

Knowledge of the unique risks that alcohol poses for women—including an increased likelihood of memory blackouts and hangovers and a faster progression of liver disease and AUD—makes recent increases in alcohol use by women more concerning.^77^ Although alcohol use by pregnant women has declined, research regarding the impact of prenatal alcohol exposure has accelerated and suggests that relatively small amounts of alcohol can produce detectable changes in morphology and deficits in cognitive and motor function. It is important to consider the unique factors that might influence alcohol use among women, and the unique direct and secondhand health effects that alcohol poses for women, when developing prevention strategies to address alcohol use and related harms.

## Figures and Tables

**Table 1:** Percentage of Past-Month Alcohol Consumption and Binge Drinking (4+/5+) and Past-Year DSM-IV AUD Among Female and Male Adolescents and Young Adults by Race/Ethnicity, NSDUH 2018

	Females						Males					
	Ages 12–17			Ages 18–25			Ages 12–17			Ages 18–25		
Race/Ethnicity*	Drink	Binge†	AUD‡	Drink	Binge†	AUD‡	Drink	Binge†	AUD‡	Drink	Binge†	AUD‡
**Overall**	9.6	5.3	1.9	55.5	34.9	8.8	8.8	4.6	1.5	54.4	35.0	11.1
**Hispanic**	8.0	3.9	1.6	49.3	33.0	8.5	6.9	3.8	1.8	49.6	21.3	10.7
**NH Asian**	5.6	3.7	1.8	45.1	23.4	8.0	3.7	2.0	0.0	43.0	32.1	10.8
**NH AI/AN**	5.8	2.1	1.1	45.1	31.1	15.5	4.7	2.9	0.7	49.8	33.0	7.0
**NH Black**	6.3	2.9	0.5	43.7	23.0	4.4	3.6	1.7	0.9	41.2	23.6	5.8
**NH Multiple**	13.3	9.2	6.7	55.7	36.3	12.5	8.4	3.4	1.2	58.9	36.9	9.7
**NH H/OPI**	14.9	11.1	4.5	24.7	17.3	18.4	1.8	1.8	0.4	54.7	46.3	15.9
**NH White**	11.5	6.6	2.2	62.8	40.3	10.0	11.6	6.2	1.8	61.0	30.6	12.7

* **Race/ethnicity:** Hispanic, non-Hispanic (NH) Asian, NH American Indian or Alaska Native (AI/AN), NH Black, NH more than one race (NH Multiple), NH Hawaiian or other Pacific Islander (H/OPI), NH White.

† **Binge drinking:** Defined as four or more drinks on an occasion for females and five or more drinks on an occasion for males (4+/5+).

‡ **AUD:** Either DSM-IV alcohol abuse or alcohol dependence.

*Source:* SAMHSA, 2019.^19^

**Table 2:** Binge Drinking Levels in the Past Year Among Women and Men Based on Sexual Identity, National Epidemiologic Survey on Alcohol and Related Conditions III, 2012–2013

	Women (%)			Men (%)		
Binge Level*	Heterosexual	Lesbian	Bisexual	Heterosexual	Gay	Bisexual
**4+/5+**	26.3	48.6	58.5	39.3	46.5	47.0
**8+/10+**	7.2	20.7	21.1	18.4	17.8	26.4
**12+/15+**	2.9	8.2	7.8	7.1	8.2	11.0

* **Binge drinking:** Defined as four or more drinks on an occasion for females and five or more drinks on an occasion for males (4+/5+).

*Source:* Adapted from Fish, 2019.^35^
